# Advanced optical microscopy methods for *in situ* single-molecule studies of membrane proteins

**DOI:** 10.1007/s12551-025-01372-0

**Published:** 2025-10-25

**Authors:** Shannan Foylan, Gail McConnell, Gwyn W. Gould

**Affiliations:** Strathclyde Institute for Pharmacy and Biomedical Sciences, University of Strathclyde, Glasgow, UK

**Keywords:** Optical microscopy, Membrane proteins, Plasma membrane, Single molecule localisation microscopy, Fluorescence resonance energy transfer, Single Particle Tracking

## Abstract

Integral membrane proteins are crucial molecules ubiquitous to all cell types, coordinating cell signalling and facilitating the tightly regulated transport of essential nutrients across plasma membrane. Defects in membrane proteins are associated with disease, emphasising the need to understand the structural, mechanistic and regulatory mechanisms which control integral membrane proteins. Recent technological advances in optical microscopy have allowed appropriate study of these small proteins using tools with molecular resolution which can non-invasively observe their native organisation in the plasma membrane in situ*.* Complimentarily, by utilising photochemical phenomena and analyses, single-molecule detail can be elucidated from conventional microscope systems. In this review, we firstly overview the methodologies used for studies of membrane proteins and then review the biophysical results gleaned from their application with an emphasis on membrane transporters. We show that single molecule studies of integral membrane proteins are beginning to unveil striking new regulatory mechanisms with wide applicability across many distinct fields of biological research.

## Introduction

The plasma membrane (PM) is of crucial importance to life, forming the boundary between a cell and the extracellular environment, thereby controlling the flow of information, nutrients and energy. While the two-molecule thick lipid bilayer provides the structural integrity of the PM, the role of mediating almost all transmembrane functions of the membrane falls to membrane proteins. These include integral membrane proteins either constitutively localised to the PM, or which translocate to the PM in response to a physiological stimulus (Alberts et al. 2014). These integral membrane proteins are responsible for a range of functions, including synthesis of adenosine triphosphate (ATP) (Nirody et al. 2020), producing or transmitting electrical signals (in nerve and muscle cells), driving active transport of specific molecules or acting as a sensor for external stimuli (Alberts et al. 2014). Recent years have seen a burgeoning understanding of the molecular details which underpin these functions.

Approximately 30% of proteins encoded in the animal genome are membrane proteins (Alberts et al. 2014; Leake 2013). Of this large subgroup, one can further define a category of membrane transporter (MT) proteins which, as the name suggests, allow transport of solutes across the tightly structured PM in a regulated manner. This family of molecular transporters are necessary to allow uptake of essential nutrients and metabolic molecules (Klip et al. 2019), excrete waste (Zepernick et al. 2024) and maintain homeostasis in intracellular ion concentrations (Alberts et al. 2014). MTs function to allow transport of their chosen substrate down a concentration gradient or couple the accumulation of a substrate against a chemical gradient to the transmembrane movement of ions, or hydrolysis of ATP, and usually involve a conformational change in the transmembrane movement of the solute. For recent reviews of membrane transporter structure and function, see Hediger et al. (2004) and Miller et al. (2025). MTs have also gained considerable interest within the biotechnology and pharmaceutical industry as potential drug delivery systems as many are poly-specific—meaning that they can transport a range of substrates with varying chemical and physical properties (Hediger et al. 2004). MTs are found in all cell types, and the function of transporting cargo is broadly similar regardless of the cell type in question. However, dysregulation of MTs can have a significant impact on whole body physiology; e.g. defective regulation of glucose transporter-4 (GLUT4) is a key driver of insulin resistance and type-2 diabetes (Klip et al. 2019), and a failure in neuronal MTs has been shown to impair cognitive function and/or be linked to neurodegenerative disorders (Kiral et al. 2018).

The function of MTs can be studied using the standard array of the biochemist’s toolbox: transport assays (on cell populations or reconstituted systems), structural studies (of highly purified proteins) or cell biological approaches (e.g. knockout or knockdown cell lines). These powerful and informative approaches have driven considerable biological insight, but are in general restricted to population studies, and by contrast, studies of purified proteins lack the spatial context of the protein in situ. MTs are generally small molecules: for example, the well-studied glucose transporter in adipocytes and myocytes, GLUT4, is ~ 15 nm in diameter with a 55-kDa molecular weight (Klip et al. 2019; Yuan et al. 2022). Therefore, appropriate study of the organisation and clustering of GLUT4 or other MTs in the PM of their respective cell types require methodologies capable of molecular resolution.

Historically, the study of structures at this scale has been in the remit of electron microscopy (EM). EM techniques require vigorous, often destructive preparation techniques including fixation, dehydration, mechanical sectioning and embedding in non-biological media, and as such, examining biological structure and dynamics in vivo is not possible with EM. Further, while EM methods have an unmatched spatial resolution in the field of microscopy, labelling structures for these methods use either heavy metals or negative staining and therefore do not have the molecular specificity available to fluorescence microscopy techniques (Lakowicz 2006b). Correlative approaches have allowed for fluorescence imaging and EM to be performed in tandem (Kukulski et al. 2011); however, these processes are generally not trivial, and so organelle specific imaging with EM is often performed with histochemistry.

In this review, we will discuss the application of advanced fluorescence microscopy techniques to the study of MTs and selected integral membrane proteins in situ in the PM. These methods strike the balance of sufficient resolution, achieve single molecule specificity and maintain membrane architecture for appropriate study of MTs in their native environment. These studies span various MTs across different kingdoms of biology, highlighting the broad applicability of these advanced biophysical methods. This review is split into two sections, the first covering the application of optical methods yielding molecular resolution and the second those where molecular resolution arises from the photochemistry of the specimens under investigation.

## Section 1: understanding single-molecule localisation microscopies

Optical light microscopy utilises illumination with a light source (wavelength ranging between 400 and 700 nm) and a series of glass lenses to form a magnified image of a specimen of interest (Davidson and Abramowitz 2002). While the insights optical microscopy has provided in scientific study cannot be understated (see Davidson and Abramowitz 2002; Thorn 2016), the spatial limit of conventional optical microscopy is set by the wave nature of the illuminating light. A single point object illuminated and imaged by a light wave cannot be imaged as a perfect point object, but rather as a diffraction pattern of the wave fronts focused by the microscope optics to form a so-called Airy disk. If this point object is illuminated by a light source of a wavelength of 500 nm imaged by a precisely aligned microscope with a high-quality objective lens of numerical aperture 1.5, the resulting image is an Airy disk diffraction pattern of the point object where the Smallest feature resolved will be on the order of 200nm, regardless of the original size of the object—this is known as the diffraction limit. Recently, a family of techniques have emerged which utilise phenomena in optical physics and photochemistry to elucidate detail of biological structures and dynamic processes below this limit. This microscopy niche, broadly known as super-resolution optical microscopy or nanoscopy (Schermelleh et al. 2019), has evolved into a research field in its own right. There are several excellent review articles which discuss super-resolution optical microscopy in depth (Schermelleh et al. 2019; Valli et al. 2021; Sahl et al. 2017); only those yielding a spatial resolution at the single molecule level are described here.

**Fig. 1:**
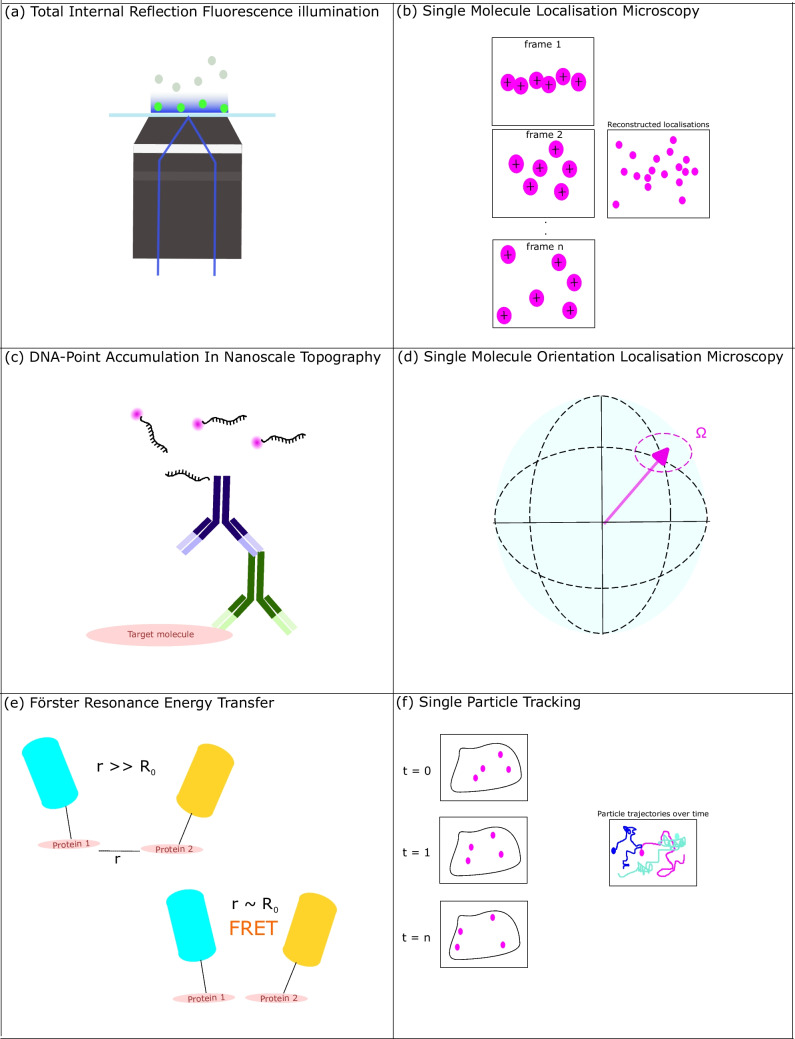
Simplified schematic representation of advanced optical microscopy methods discussed in this review. **a** Total Internal Reflection of excitation light (blue) resulting in Fluorescence of fluorophores within the ~ 100 nm evanescent field depth of a TIRF objective. **b** Single-molecule reconstruction across temporarily separated acquisition frames in dSTORM imaging. **c** DNA-oligomer labelling protocol for DNA-Point Accumulation In Nanoscale Topography imaging where fluorescence blinking event arises from the transient binding of single stranded ‘imager’ strands in solution to single stranded ‘docking’ strand conjugated to target molecule, usually an antibody against a specific target protein (**d**) measuring the Ω (‘wobble’) of a molecule of interest using the polarisation of the dipole moment of the molecule in Single Molecule Orientation Localisation Microscopy, Poincaré sphere in blue indicating the polarisation of the molecule of interest. **e** Förster (Fluorescence) Resonance Energy Transfer of proteins within characteristic Förster radius. **f** Single-Particle Tracking of fluorescently labelled targets of interest over time

Iterative optical microscopy techniques which allow for localisation of single molecules rely on photochemical switching of fluorescent labels such that only a sparse subset of labelled structures is acquired in a single image frame. When acquiring over several thousand (generally on the order of 10,000—20,000 (Lelek et al. 2021)) frames, every fluorophore will be excited and captured but in a temporally separate manner, allowing the diffraction limit to be overcome. This family of techniques is known as single-molecule localisation microscopy (SMLM) methods and includes stochastic optical reconstruction microscopy (STORM) (Rust et al. 2006; Henriques et al. 2011) (laterally direct(d)-STORM (Linde et al. 2011) where ‘direct’ denotes fluorescent antibody pairs not being required), photo-activated localisation microscopy (PALM) (Betzig et al. 2006) and point accumulation in nanoscale topography (PAINT) (Sharonov and Hochstrasser 2006) (laterally DNA-PAINT (Schnitzbauer et al. 2017) utilising DNA oligomers). These are outlined further below; each brings a different set of advantages, and all have been used in studies of MTs which we elaborate upon further in the “Section 2: single-molecule localisation microscopy enabled studies of MTs in situ” section of the review. A schematic overview of all methods discussed is provided in Fig. 1 for clarity.

Total internal reflection fluorescence (Axelrod 1981): All SMLM techniques require a sufficiently high signal-to-background ratio (SBR) to elucidate these single molecule blinking events. A common means of improving SBR is via total internal reflection fluorescence (TIRF) illumination (Fig. 1a). TIRF introduces the illumination source at an angle above the super-critical angle at the interface between the refractive index of the specimen under examination and the glass substrate on which it is being imaged (Fig. 1a). This restricts fluorescence excitation to a single optical section with a thickness on the order of 100 nm, allowing imaging of membrane structures within a few hundred nanometres of the boundary of the cell. TIRF is a microscopy method in its own right and has been utilised extensively in cell biology to image cell contacts (Axelrod 1981) to study adhesion of vesicles to the PM (Cardoso Dos Santos et al. 2016; Oreopoulos and Yip 2009; Ward et al. 2020), to study ion channels (Luik et al. 2006) and endocytosis (Taylor et al. 2011) and to examine the self-assembly of proteins into peptide chains (Bella et al. 2014).

dSTORM (Linde et al. 2011) relies on labelling with conventional fluorophores but uses a chemical buffer to switch the molecules to the dark/off state in a reliable, reversible manner (Fig. 1b). TIRF illumination with a laser of appropriate wavelength and power switches a small subset of the total fluorophore population on and fluorescence emission is recorded with a widefield microscope. By acquiring over several thousand image frames in rapid succession, individual fluorophores are switched to an on state, temporally separate from each other, allowing computational reconstruction of the full field image to identify blinking events from background noise. The computational localisation of these blinking events relies on fitting a Gaussian curve to each object and determining the peak intensity. The localisation precision of a system scales as a function of the point spread function of the objective lens used and is inversely proportional to the square root of the number of photons collected (Lelek et al. 2021). As such, localisation precision and the associated optical resolutions achieved in dSTORM are an order of magnitude below the diffraction limit. dSTORM systems have reported the ability to resolve structures down to 20 nm in size, with a variety of optimised switching buffers presented by different researchers (Goossen-Schmidt et al. 2020; Wang et al. 2024). However, due to the long acquisition times required for a sufficient reconstruction of specimens, dSTORM is only suitable for fixed specimens (Linde et al. 2011). Controlling photoswitching performance is key for SMLM in quantitative biology, and key metrics such as spot brightness, the fluorescent on-state lifetime (τ on) and off-state lifetimes should be optimised for a range of different buffer settings (Herdly et al. 2023) to enhance the range of data captured. Further, dSTORM has no control over which subset of fluorophores are switched on, meaning that the localisations of two structures of interest which lie within the area of overlap of their two reporting antibodies are indistinguishable from each other—a phenomenon known as the antibody linkage error (Ries et al. 2012).

PALM (Betzig et al. 2006) is functionally similar to dSTORM (Fig. 1b) insofar as the hardware consists of a widefield microscope with TIRF illumination and a computational reconstruction of fluorescence blinking events is made to improve spatial resolution of the image. The difference between PALM and dSTORM relies on the means of generating the ON/OFF blinking of the reporting fluorophores for localisation. PALM relies on a family of photoactivatable fluorophores which are subsequently bleached following imaging. The ON state is induced by a (generally spectrally separate) wavelength of light, and the OFF state is induced by forcing the excited electron into the dark state. The family of photoactivable fluorophores utilised by PALM has expanded significantly since the initial demonstration of the technique (Henriques et al. 2011).

PAINT (Sharonov and Hochstrasser 2006) relies on the transient binding and unbinding of fluorescent ligands to generate the blinking events necessary for a SMLM reconstruction. The most commonly employed method, DNA-PAINT (Schnitzbauer et al. 2017) (Fig. 1c), utilises complementary DNA oligomers, the ‘docking’ strand of which is conjugated to a biomolecule of interest and the ‘imaging’ strand is conjugated to the reporting fluorophore which will fluoresce upon base pair binding of the two strands. Unlike dSTORM, DNA-PAINT does not require specialised switching buffers, and the nature of transient binding to ‘DNA barcoded’ targets allows for simplified multi-colour imaging with a single excitation wavelength. The multiplexing ability of DNA-PAINT has dramatically expanded in recent years (Jungmann et al. 2014; Chung et al. 2022; Reinhardt et al. 2023) allowing for imaging down to the 5-nm range (Chung et al. 2022) or even Angstrom scale (Reinhardt et al. 2023). Further, by developing hardware to perfuse imager strands into and out of a DNA-PAINT specimen, researchers have shown the capability of the method to move towards high-throughput or multiplexed acquisitions while automatically removing any unbound imager strands (35, Tinning et al. unpublished results). There are, however, some limitations of these impressive methods. As with its counterpart in dSTORM, DNA-PAINT requires several thousand image frames for accurate localisation, making it best suited for fixed specimen imaging. However, recently, LIVE-PAINT (Oi et al. 2020) has been demonstrated to somewhat obviate this limitation but is still unsuitable for the study of highly dynamic specimens. Oi et al. comment that when studying dynamic actin structures, the number of localisations detected in a 200-s imaging window decreased and some known actin structures are not visible with TIRF illumination (Oi et al. 2020). Availability of antibodies and fluorophores conjugated to DNA oligomers for these methods is growing but is by no means as ubiquitous as conventional antibodies for diffraction-limited immunofluorescence. A further issue which is compounded upon multiplexing is that DNA-PAINT labelling is an expensive endeavour, requiring a specifically designed docking oligomer strand to the target of interest, with a biotin-NeutrAvidin complex separating the labelling oligomer from the target, alongside a complementary base pair binding DNA imager strand conjugated to a reporting fluorophore for single colour DNA-PAINT imaging (Schnitzbauer et al. 2017).

Single-molecule orientation localisation microscopy (SMOLM) is a multidimensional variant of SMLM where spatial position and molecular orientation are encoded into widefield image data (Fig. 1 d). This method provides detail on the organisation of the structure under study alongside rotational diffusion information on the molecule, describing how the molecule ‘wobbles’ in its environment. SMOLM relies on recording the phase and polarisation alongside conventional fluorescence intensity (Zhang and Lew 2024). While the multiparameter benefits of SMOLM are clear, the optical setups required are more complex (and indeed more expensive with the requirement of polarisation-sensitive detectors) than intensity-based SMLM systems, and the post-processing required to extract molecular rotation values is non-trivial.

## Section 2: single-molecule localisation microscopy enabled studies of MTs in situ

In the “Section 1: Understanding Single-Molecule Localisation Microscopies” section, we provided a birds-eye view of different SMLM approaches. Here, we consider exemplar studies in which these approaches have been applied to the study of membrane proteins and provide some context to the novel information these studies have provided. Often, these advances are methodological: SMLM approaches can be prohibitively complex to the non-expert and experiments involve expensive reagents. Hence, our synopsis below includes both advances in instrumentation and analysis, in tandem with new biological insight driven by these methodological steps. We consider two types of studies—those which identify clustering behaviour of integral membrane proteins within membranes as a regulatory mechanism (Sect. ‘Identifying and quantifying nanodomains within the plasma membrane’), and studies which directly study membrane transporter proteins and their behaviour at single-molecule levels (Sect. ‘Resolving new regulatory mechanisms for membrane transporters’). In the data we have chosen to highlight as figures in this review, we focus on novel insights UK and Eire-based researchers have gleaned from MT of interest using advanced optical microscopy methods for the purposes of this review series. Readers are referred to a range of recent reviews to facilitate a further appreciation of the wide array of applications of SMLM across other experimental systems (Sharrocks et al. 2025; Radmacher et al. 2025; Zhang and Lew 2024; Steves et al. 2024; Barsanti et al. 2023; Brenner et al. 2023; Modak et al. 2024).

### Identifying and quantifying nanodomains within the plasma membrane

The eukaryotic PM contains domains of varying lipid composition and biophysical properties (Sezgin et al. 2017; Levental and Lyman 2023). These domains are below the resolution limit of optical microscopy and thus are difficult to study, and hence, understanding protein localisation to or exclusion from these domains and the associated dynamics has proven challenging.

#### Studying the biophysical landscape of the PM

**Fig. 2:**
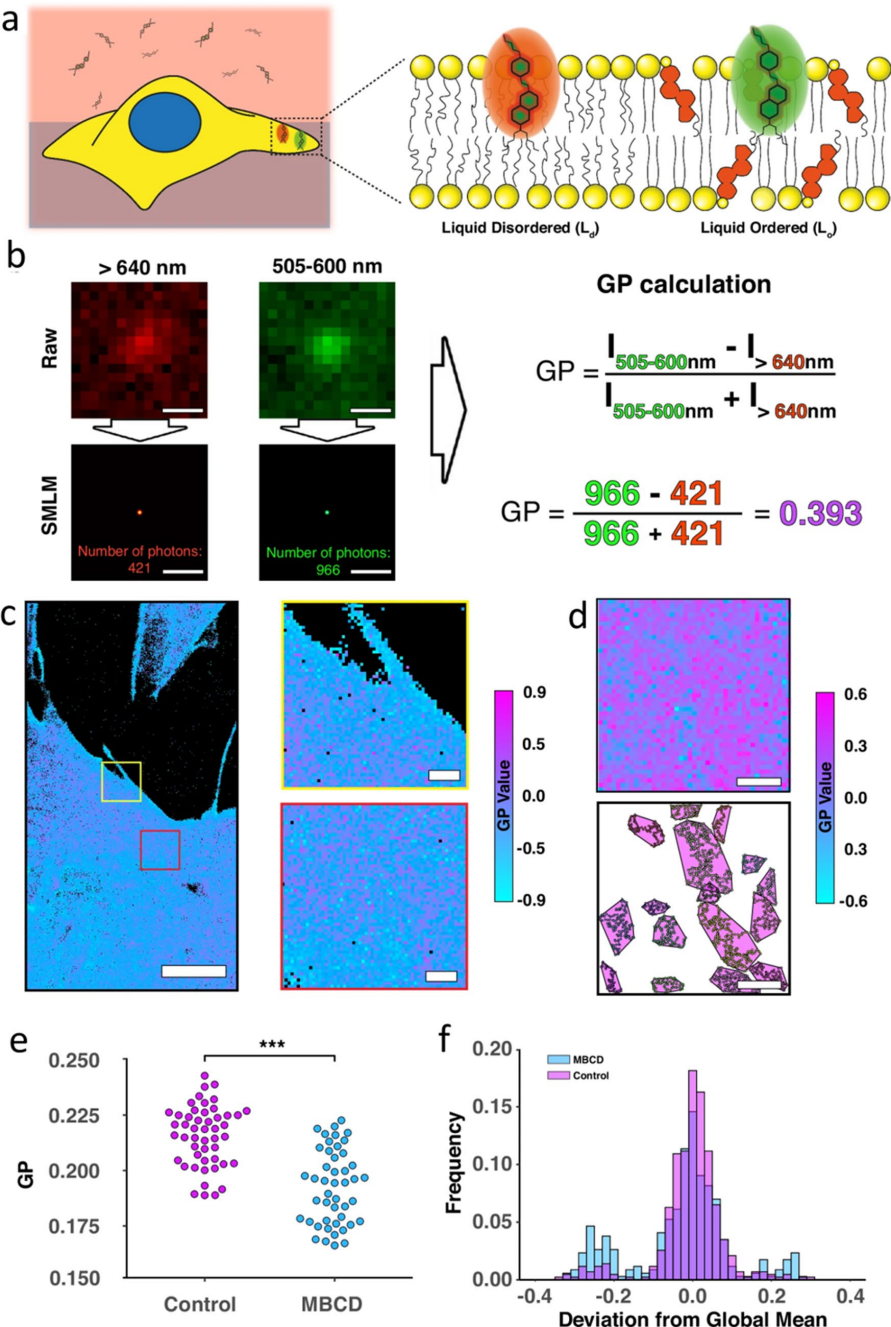
Local polarisation measurements on single membrane proteins. **a** Schematic of solvatochromic probe principle in Panconi et al. (2024), di-4-ANEPPDHQ probes diffuse rapidly in solution and bind to the PM upon contact and their fluorescence emission is altered by their polarity, shown here as liquid-ordered and liquid-disordered membranes. **b** Generalised polarisation values from experimental ratiometric di-4-ANEPPDHQ PAINT data. PSFs localised from SMLM image and photon counts at each coordinate yield GP value. Scale bars 500 nm. **c** Representative image of fibroblast membranes from an 8-min acquisition with two highlighted regions of interest, with GP colour scale (black indicating no localisation detections). **d** Exemplar data of di-4-ANEPPDHQ PAINT live cell data, segmented by averaging GP to obtain regions or high and low order. **d** Average GP value for all points within selected ROIs for untreated cells (Control, magenta) and cells treated with the cholesterol-sequestering agent methyl-β-cyclodextrin (MβCD, cyan). MβCD treatment caused a significant decrease in GP. **e** Histogram of the frequency of identified domains fell into intervals above (ordered) or below (disordered) the global average GP value (here, ~ 0.22 for untreated control and ~ 0.17 for MβCD) for both the untreated cells (control, magenta) and those treated with MβCD (cyan). Scale bars: **c** 5 μm in left hand large FOV, and 500 nm in **d**). Figure reproduced from Panconi et al. (2024) with permission https://creativecommons.org/licenses/by/4.0/

Panconi et al. (2024) recently outlined one route to circumvent domain confusion: a combination of a solvatochromic fluorescent probe with SMLM in order to elucidate localisation coordinates of a protein of interest in PM domains in tandem with information on the local polarisation of the environment of the probe. The authors employ PAINT imaging, firstly of Giant Unilamellar Vesicles and subsequently of rat fibroblasts labelled using the di-4-ANEPPDHQ probe (which reports on its biophysical environment via spectral changes in fluorescence emission) in conjunction with open-source software to map the nanodomains revealed by said SMLM imaging. This approach yields a generalised polarisation (GP) value which infers a measure of order in specific nanodomains of the membrane under study (high GP indicates an area of order and low GP an area of disorder) which is overlain on the PAINT fluorescence intensity image as an intuitive read of membrane order (see Fig. 2) to allow identification and a focus on key areas of interest within the PM.

This recent development of an experimental pipeline employing a commercially available potentiometric probe in conjunction with SMLM and the analysis and visualisation tool presented by the authors demonstrates a novel tool for studying the biophysical landscape of the complex plasma membrane under different physiological conditions. This is an important step, as the role of nanodomains in mediating many functional aspects of the PM remains a controversial subject. These and similar approaches will begin to deliver clarity on these issues.

#### Understanding Wegovy

Buenaventura et al. (2019) studied the influence of the local lipid environment in the PM on the trafficking and functionality of the type-2 diabetes implicated glucagon-like peptide-1 (GLP-1) receptor (GLP-1R) in pancreatic beta cells. Using an impressive combination of conventional confocal microscopy, PALM, EM and fluorescence resonance energy transfer (FRET) (discussed in detail in the “Section 3: obtaining molecular resolution from a diffraction-limited microscope” section) imaging modalities in conjunction with palmitoylation and insulin secretion assays, it was found that GLP-1R associates with liquid-ordered PM nanodomains in a palmitoylation-dependent manner, and that the average number of GLP-1 molecules in these clusters increases upon stimulation with the GLP-1R agonist extendin-4. This is proposed to create ‘hotspots’ which act to coordinate signalling and clathrin-dependent endocytosis of the GLP-1R, hence fine-tuning cellular responses. After stimulation with different GLP-1R agonists and modulating posttranslational modifications of the receptor, the authors observed a marked difference in GLP-1R clustering, indicating that the lipid architecture of the PM could be a potential further target for therapeutics for dysregulated GLP-1 signalling. The potential importance of such studies is underscored by the recent boom in the use of GLP-1R agonists in regulating body weight as a therapeutic tool.

#### Instant gratification? POLCAM: instant molecular orientation microscopy for the life sciences

**Fig. 3:**
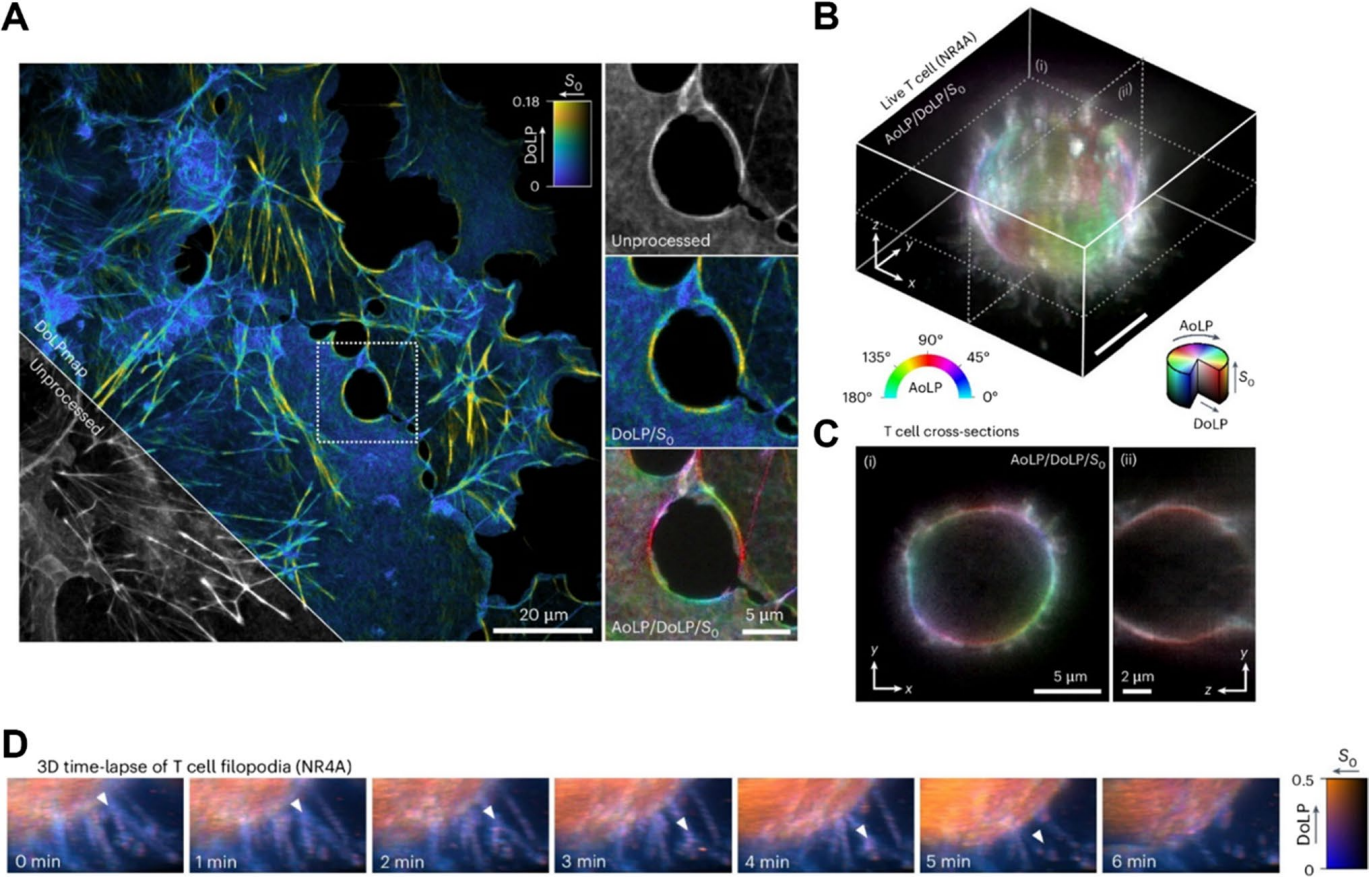
Diffraction-limited polarisation microscopy using POLCAM. Shown is data from Bruggeman et al. (2024) which uses POLCAM, a simplified SMOLM method based on polarised detection using a polarisation camera, implemented on a widefield fluorescence microscope. **A** Diffraction-limited polarisation camera image of fixed COS-7 cells labelled with SiR–actin, rendered using a colour map to show the degree of linear polarisation (DoLP). An inset marked by a dotted square is shown in an unprocessed, DoLP colour map and an HSP polarisation colour map. **B** A 3D image of the plasma membrane of living T cells using the membrane polarity probe NR4A. **C** Two cross-sections of the T cell shown in B. **D** A 3D timelapse of the movement of the filopodia of a live T cell rendered using a DoLP color map, illustrating the power of this approach in living cells. Randomly polarised regions appear blue (filopodia) and more structured, and therefore, polarised areas (larger, more smooth sections of the plasma membrane surface) appear orange. A white triangle tracks the movement of what appears to be a branching point on a filopodium. Reproduced from Bruggeman et al. (2024) under the terms of the Creative Commons Licence CC BY

Single-molecule orientation localisation microscopy (SMOLM) encompasses high precision localisation coordinates with an analysis of the orientation of individual fluorescent molecules; this in turn can offer insight into how these molecules are arranged and move within their environment. Although powerful, the application of SMOLM is limited in part by the complexity of the experimental system and in part by the complex post-image acquisition analysis. Bruggeman et al. (2024) developed and demonstrated a simplified experimental pipeline for achieving single-molecule spatial localisation and orientation resolution, shown in both a diffraction-limited system and a SMLM system. By the simple addition of a polarisation camera (with a directly integrated polariser on the camera sensor chip), the authors demonstrated the ability of their technique, POLCAM, to achieve a 7.5° orientation angle precision alongside a 5-nm localisation precision in model membrane systems. The authors present the biological applicability of their experimental setup via dSTORM reconstructions of actin fibres in HeLa cells and widefield orientation-resolved images of plasma membrane labelled T cells (Fig. 3). These studies clearly exemplify the potential advances in both speed and imaging hardware simplicity that this approach can offer. Further, the authors developed software to analyse the polarisation encoded acquisitions to resolve orientation values in 3D and account for known artefacts from imaging single molecules with a polarisation-sensitive camera. Simplified SMOLM imaging presents the field with a novel tool to simultaneously measure both spatial localisation and orientation of a labelled protein in the PM. The caveat being the range of labelling protocols which immobilise or control the orientation of an imaged fluorophore remains limited.

#### Surface clustering reveals regulatory steps in endocytosis at nerve terminals

Small et al. (2024) utilise single-molecule imaging to study the nanoscale organisation of synaptic vesicular proteins at the plasma membrane of isolated primary hippocampal neurons. A key facet of neuronal cell biology is the exocytosis of small synaptic vesicles to the PM where they dock and fuse and release their content (neurotransmitters) into the synaptic cleft. Key to this process is the ability to rapidly and specifically retrieve synaptic integral membrane proteins from the PM and reinternalise/recycle them for synaptic vesicle recycling. The authors explore the complex interaction between two synaptic vesicle proteins—synatotogamin-1 (Syt1) and synaptic vesicle protein 2 A (SV2A)—using PAINT microscopy. Their studies revealed that these two proteins form presynaptic nanodomains, discrete from clathrin endocytic machinery, and that there was an enrichment of Syt1 at the nerve terminals. By knocking down SVA2 before imaging, it was observed that the mobility of Syt1 significantly increases in the absence of the regulator SVA2. By combining their imaging with electrophysiological patch-clamping experiments, the authors found that the recycling function of SVA2 is unimpacted by Syt1 but that the loss of interaction with SVA2 causes a host of issues for Syt1, including diminished number of PM nanoclusters, smaller area and shorter lifetime of remaining nanoclusters and an increase in molecular mis-sorting via the Syt1 pathway. The insight revealed by the single-molecule imaging approaches utilised in this study argues that the molecular interaction between Syt1 and SV2A drives the formation of nanoclusters, and that these clusters act upstream of endocytosis, thereby serving to control the availability of Syt1 for endocytic retrieval. Thus, it is tempting to suggest that these newly identified nanoclusters can titrate the rate of recycling of Sty1 during neurotransmission. This may turn out to be a key regulator for other PM proteins.

### Resolving new regulatory mechanisms for membrane transporters

#### Aquaporin clustering in astrocytes

Aquaporins (AQP) are a family of MTs which facilitate water movement into and out of a cell driven by the osmotic gradient; aquaporin-4 (AQP4) predominates in the membrane of astrocytes. AQP4 has been shown to be critical in the regulation of cerebrospinal fluid circulation and clearing waste, acting as a neuroprotective molecule against traumatic brain injury and ischemic stroke (Hussain et al. 2023; Monai et al. 2019). EM studies have revealed a striking clustering of the aquaporin-4 isoform (AQP4) in astrocytes, a sub-type of glial cells that predominate in the human central nervous system but the functional relevance of these clusters remained obscure.

**Fig. 4:**
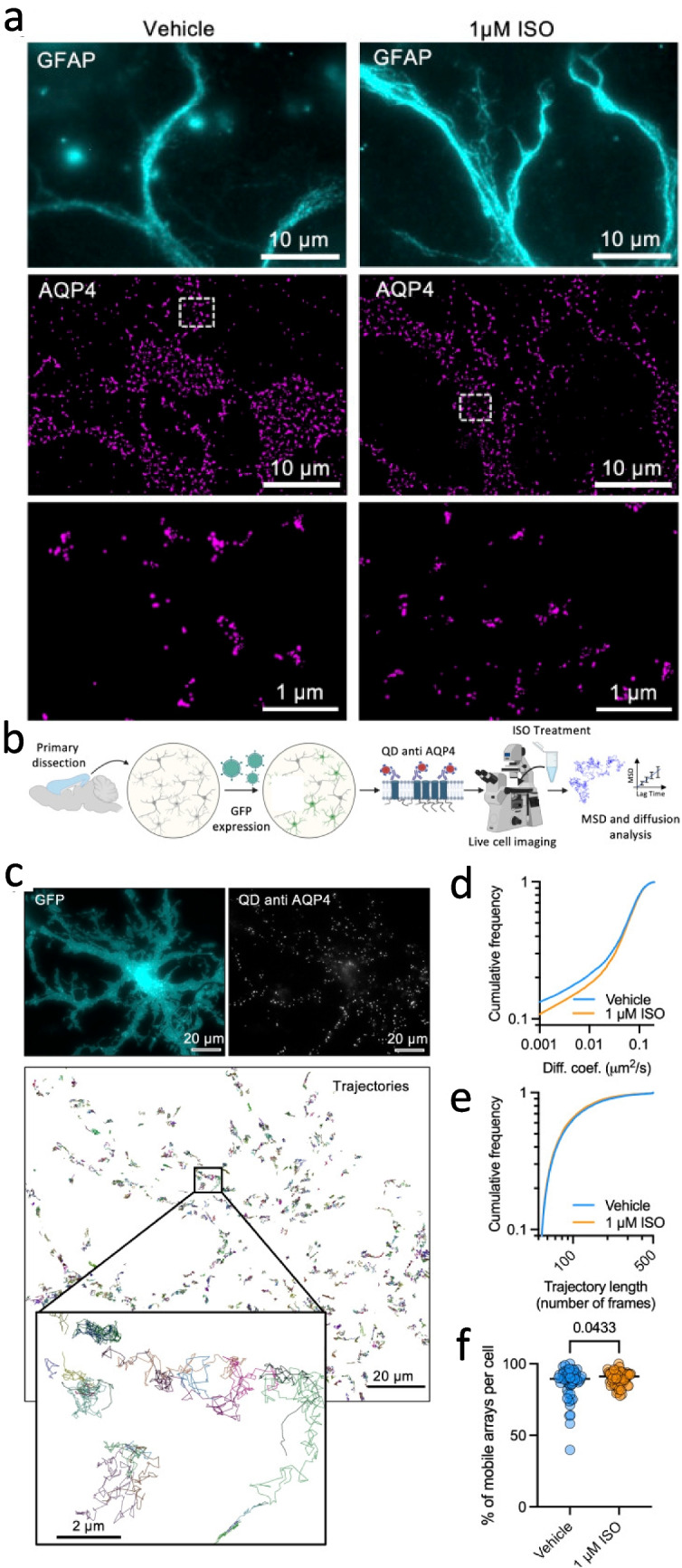
Revealing the structure and function of neuronal water transporters with SMLM. dSTORM imaging elucidates adrenergic signalling as a regulator for aquaporin-4 array size. **a** (top panel) Representative TIRF images of cortical astrocytes stained for glial fibrillary acid protein (GFAP, cyan) and treated with isoproterenol (centre panel) corresponding dSTORM images of AQP4 arrays (magenta) rendering localisations with dots of sizes corresponding to the respective localisation precision, (lower panel) region of interest from dSTORM image. **b** Experimental workflow for quantum dot tracking (created with BioRender). **c** Representative images of cortical astrocytes expressing GFP, labelled with quantum dots functionalised against aquaporin-4 with reconstructed trajectories. **d** Cumulative distributions of the diffusion coefficients of aquaporin-4 (mean ± SD) in vehicle control conditions (*n* = 55 cells) and in response to 1 μM ISO (*n* = 56 cells). **e** Cumulative distributions of trajectory lengths (number of frames) for both experimental conditions. Diffusion data was obtained from five independent experiments (Kolmogorov–Smirnov test: *D* = 0.03431, *p* < 0.0001). **f** Percentage of mobile aquaporin-4 arrays per cell for the same experiments shown in (c) and (d), showing an increase in the proportion of mobile vs. immobile arrays upon isoproterenol treatment (unpaired *t*-test, *p* = 0.0433). Reproduced from Zepernick et al. (2024) under the terms of Creative Commons Attribution 3.0 Unported Licence per the Royal Society of Chemistry

Zepernick et al. (2024) tested the hypothesis that an activation of beta-adrenergic receptors regulates both the size and dynamic properties of AQP4 via the osmotic swelling of astrocytes. Due to the nanoscale organisation and sub-micron architecture of the surrounding environment, the authors utilised dSTORM imaging of both fixed and live isolated murine astrocytes. From these studies, it was observed that the beta-adrenergic receptor agonist isoproterenol both decreased the size of AQP4 clusters and increased their mobility in the PM (see Fig. 4). This result was corroborated with live imaging of the extracellular domain of AQP4 labelled with a low density of functionalised quantum dots, where single-particle tracking (illustrated schematically in Fig. 1f) revealed a decrease in mobility for isoproterenol-treated AQP4 molecules.

Complimentarily, the authors presented a means of reversing the effects of isoproternol by a simple change of tonicity in the cell culture medium to introduce an osmotic change to the cells; this in turn led to an increased cluster size and number of localisation events. This demonstrates a novel physiological regulatory mechanism for AQP4 size and mobility, in live cells with endogenous labelling, providing possible therapeutic avenues for treating ineffective waste clearance in astrocyte cells.

#### Clustering of glucose transporters

Glucose is the primary source of energy for cells, and as such, regulation of blood glucose is of vital importance to health. The GLUTs, a family of 14 glucose MTs, are responsible for glucose transport in humans. The most studied member of this family, GLUT4, is responsible for insulin-stimulated uptake of glucose into fat and muscle tissues; GLUT4 exhibits regulated delivery to the cell surface in response to insulin, and dysregulation of this process has been shown to be a hallmark for insulin resistance and type-2 diabetes (Klip et al. 2019). TIRF microscopy has revealed two distinct organisations for GLUT4 within the PM of fat cells—clustered in basal cells and dispersed in insulin-stimulated cells (Stenkula et al. 2010). Using dSTORM imaging, Koester et al. (2022) corroborated the insulin-dependent dispersal of GLUT4 and further showed that the scaffold protein EFR3 and phosphatidylinositol PI4-kinase IIIα are necessary for insulin-stimulated glucose transport in fat cells. The authors observed that while knockdown of EFR3 hindered the dispersal of GLUT4 in response to insulin, the insulin-stimulated levels of GLUT4 at the cell surface remained unchanged. These results indicate that translocation of GLUT4 to the cell surface is not hindered in the absence of EFR3 and suggest that EFR3 plays a role in post-PM delivery dispersal of GLUT4 within the PM. Importantly, these data showed that glucose transport is impaired upon EFR3 knockdown, suggesting a functional link between GLUT4 transport activity and its clustered/dispersed status.

The first member of this glucose transporter family to be characterised, GLUT1, is responsible for uptake in neuronal tissues and transporting glucose across the blood–brain barrier. Overexpression of this MT is an important hypoxic marker in tumours and is used in prognosis of tumour progression. However, the structure and organisation of GLUT1 in the PM have been under debate with different researchers suggesting different quaternary structures based on kinetic models and co- or overexpression studies (Cura and Carruthers 2012). Yan et al. (2018) employed dSTORM imaging to image both the clustering of GLUT1 in the PM and its colocalisation with lipid rafts in situ. The authors observed average cluster sizes sitting around the diffraction limit but that the cluster sizes were on average smaller on the media exposed side of the cell relative to the basolateral side. Cluster size was shown to be variable, but those with two to four molecules were shown to be average (however, this data should be caveated with the antibody linkage issue noted above). By employing two-colour imaging, the authors observed that 35% of the imaged GLUT1 were associated with lipid rafts and that disrupting the formation of the rafts hindered clustering. Further, depolymerisation of the actin cytoskeleton and hindering glycosylation were shown to disrupt the clustering of GLUT1 but did not affect the overall amount of the molecule in the PM. In this instance, two-colour dSTORM imaging allowed the authors to elucidate key organisation features of this crucial MT in the PM of HEK293 cells.

The signalling cascade which triggers trafficking of GLUT molecules to the PM to uptake glucose begins with the insulin receptor (IR). As such, the dysregulation of this molecular pathway, which is indicative of metabolic disorders, is inextricably linked to the dynamics and organisation of the IR within the PM. Dall’Agnese et al. (2022) utilised time-correlated PALM (which couples the high spatial resolution of SMLM with time-correlated single photon counting electronics and analysis methods to infer dynamics of observed molecules) to elucidate the clustering behaviour of the IR in the PM (and cytoplasm and nucleus) of hepatocytes. The authors observed a varied dynamic behaviour of these clusters but that both in the presence and absence of insulin, most clusters were short-lived, exhibiting an average lifetime of between 6 and 12 s. The clusters which did not fall into this large group persisted for a longer time (> 100 s). The number of detected localisations per cluster increased upon insulin stimulation, implying multiple molecules are within a cluster which recruit more IR in the presence of insulin. In insulin-resistant cells, time-correlated PALM imaging revealed a longer persistence of clustering which was also shown to be partially reversed by treatment with the anti-diabetic drug metformin. This work presents a novel model for IR nanoscale organisation which may provide a foundation for development of therapeutics for type-2 diabetes, and should also serve as a paradigm for studies of other key PM receptors.

#### Pyroptosis studied one molecule at a time

Gasdermin proteins are responsible for initiating death programs in infected or damaged cells via pyroptosis by forming pores in the PM to recruit immune cells. Gasdermin proteins are harmless in the cytosol and require activation via cleavage by inflammation proteases. While the full cell picture is well understood, details on recruitment of these proteins to the PM and their oligomerisation within it are unknown. Margheritis et al. (2024) utilised single-molecule TIRF imaging and stoichiometric analysis of image data to study these processes, initially using a purified model membrane system which allowed careful quantification of different molecular species. This revealed that gasdermin proteins preassemble at the membrane into dimeric and trimeric ‘building blocks’. These in turn can either be inserted into the membrane or undergo a further assembly into higher-order oligomers prior to insertion. Using TIRF imaging and stoichiometric analysis, the authors were able to identify three cysteines crucial for the stepwise formation of gasdermin-induced pores in the PM. This is of considerable potential importance as gasdermin proteins have been implicated in diseases such as sepsis and viral infections and a need for effective small molecule therapeutics is evident (Zhu et al. 2024). The ability to regulate pyroptosis is therefore a promising therapeutic strategy, and the new insight garnered from these single molecule studies provides new mechanistic understanding that was notably absent from previous studies, defining key molecular assemblies in the formation of the active pores.

#### Revealing novel synaptic protein organisation with SMLM

Glycine receptors (GlyR) and gamma-aminobutyric acid type A receptors (GABAaR) are MTs responsible for mediating fast inhibition in neurotransmission and regulate the central nervous system (CNS) (Zhu et al. 2024). Previous super-resolution microscopy has revealed a ‘trans-synaptic nanocolumn’ model of protein organisation along excitatory synapses. Yang et al. (2021) employed two-colour dSTORM imaging and analysis to firstly identify that these nanocolumn structures are present in inhibitory synapses in spinal cord neurons, where spectrally separate secondary antibodies label pre- and postsynaptic proteins. By selecting nanocolumns orthogonal to the optical axis of the microscope for analysis, the authors were able to measure the distance between the pre- and postsynaptic proteins at inhibitory synapses to be on the order of ~ 100 nm. Upon confirming the existence of the structures in CNS neurons, they labelled GlyR and GABAaR and utilised two-colour dSTORM to study the recruitment of these proteins to the synapse, their organisation and to ascertain if this native organisation was altered by neuronal activity. Colocalisation analysis demonstrated that GlyR and GABAaR exist as discrete clusters, and that altering the activity of the neuronal network pharmacologically only affected the distribution of GABAaR and not GlyR, both across the synapse and at the sub-synaptic level. This SMLM work reveals both structural and functional detail on these receptors in inhibitory synapses which may overall affect the neuronal plasticity. Molecular detail is key to these advances.

#### Observing single protein oligomerisation with DCC-SMLM

Effective study of the oligomerisation of proteins generally requires disruption of the in situ organisation and interactions of the protein of interest, or a reliance on imaging modalities with insufficient resolution. Indeed, even with the high spatial precision of SMLM, a localisation of a subunit of an individual protein can lie within the localisation radius of its neighbour. To address this issue, Tan et al. (2022) present a method for dual-colour colocalisation SMLM (DCC-SMLM), where two spectrally distinct fluorophores are tagged to the same protein of interest and developed an algorithm which calculates the probability of detecting a protein subunit and the colocalisation ratio between the two fluorophores. The method corrects for sample drift inherent to the long acquisition times of SMLM, and chromatic aberration was accounted for experimentally. The authors calibrated their method with known oligomeric states of a monomeric chloride channel, dimeric chloride channel, trimeric glutamate channels and a tetrameric potassium channel before applying the method to the contested oligomerisation of the proteins SLC26 and SLC17. These MTs are responsible for transport of negatively charged ions across the PM and are implicated in a host of diseases (Alper and Sharma 2013; Reimer 2013). From this pipeline, the authors measured a dimeric state for two SLC26 proteins, the epithelial anion exchanger SLC26A3 and the motor protein Slc26a5, and a monomeric state for four SLC17 proteins (the vesicular glutamate transporters vGlut1, vGlut2 and vGlut3 and the lysosomal sialic acid transporter sialin). These hitherto unknown oligomerisation states were measured without solubilising the proteins and from populations of proteins from the widefield acquisition. This represents an exciting advance in the field of optical microscopy to reveal the intricate interactions of individual proteins with themselves.

## Section 3: obtaining molecular resolution from a diffraction-limited microscope

While obtaining spatial detail of single molecules is clearly not possible with a diffraction-limited microscope, various niches of diffraction-limited microscopy have come into being which allow for the elucidation the dynamic properties of or the interactions between single molecules, arising from either the chemistry of the molecule or its trajectory.

Förster (or fluorescence) resonance energy transfer (FRET) is a phenomenon between two proximal fluorescently labelled molecules which can be exploited to measure the distance between said molecules, elucidating context on the molecular interaction (Fig. 1e). A FRET interaction is dependent on a characteristic distance between two fluorophores which imparts energy from one (the donor) to the other (the acceptor) in a dipole–dipole interaction (Shrestha et al. 2015). Due to the strict confines of both energy exchange and the physical barrier that fluorophores too close to one another will collide, molecules undergoing FRET must be between 1 and ~ 6 nm from each other. FRET efficiency between two molecules a distance *r* (Lakowicz 2006a) apart is given by

$${E}_{FRET}= \frac{1}{1+({R}_{0})}{r}^{6}$$

where *R*_0_ is the characteristic distance between molecules where FRET efficiency drops by 50%. In an aqueous solution, *R*_0_ is given by

$$R_0=9.78\times10^3\lbrack\kappa^2n^{-4}Q_DJ(\lambda)\rbrack\;\left(\overset\circ A\right)$$

where *κ* is the orientation factor of the molecules, *Q*_D_ is the quantum yield of the donor, *n* is the refractive index of the medium and *J*(λ) is the integral of the spectral overlap—i.e. the wavelength window where a FRET interaction is possible.

In fluorescence intensity imaging, heteroFRET—that is a FRET interaction between two different fluorophores—can be observed by setting up the imaging microscope emission filters to only detect the acceptor fluorescence emission wavelength while only exciting with the fluorescence excitation wavelength of the donor. This yields an experimentally simple means of observing interacting molecules with a conventional, diffraction-limited microscope with an appropriate choice of FRET pair fluorophores. Spectroscopically, a FRET interaction is observed as a transfer of energy from acceptor to donor in the relative intensity of the Stokes paired molecules, while providing further sensitivity to detect FRET interactions between the same type of fluorophore—homoFRET. Using time-correlated single photon counting detection, FRET interactions can also be observed as a change in fluorescence lifetime between proximal FRET pairs (Duncan et al. 2004).

Single-particle tracking (SPT) allows for study of the motion of single molecules in their native environment (Fig. 1f). This technique allows the observer to interpret whether a tracked particle is undergoing Brownian motion, which is bound to an immobile structure (such as a membrane) or any changes to its interactions over an observed time period (for example, a binding or complex forming event) (Shen et al. 2017; Simon et al. 2024). In practice, SPT requires an experimental system capable of observing single particles and an analysis pipeline suitable for following the trajectories of particles over a time series. In this instance, a single particle does not necessarily correspond to a single molecule, and diffraction-limited fluorescence microscopes with much improved temporal resolution than SMLM can be used to elucidate detail on molecular dynamics in vivo (Yu et al. 2019). Coupling structured super-resolution optical microscopy techniques such as stimulated emission depletion (STED) microscopy and MINFLUX (Scheiderer et al. 2024) with SPT has shown that it is possible to infer both spatial position and molecular dynamics from live cell fluorescence time series. STED microscopy provides super-resolution detail by utilising two superimposed laser sources, namely a conventional Gaussian excitation beam and a longer wavelength masked ‘donut-shaped’ depletion beam that forces emitting fluorophores within the profile of the depletion beam into a ground state. Therefore, only fluorophores within the unmasked region of the excitation beam are detected and resolutions on the order of between 30 and 50 nm are obtained. MINFLUX (and then now combined MINSTED) approach the localisation achieved by chemical means in SMLM via an optical technique. By scanning the coupled excitation and depletion beams around the fluorophore of interest, one can infer from the recorded intensity histogram where the centre of the object lies spatially, triangulating a localisation. One can use the focus of such a system to track molecules with a high degree of spatiotemporal resolution: for example, the authors presenting their modality tracked kinesin-1 motors travelling down a microtubule. An important limitation of this methodology is that the volume of study is significantly smaller than SPT studies carried out with a widefield microscope.

## Section 4: MT studies utilising diffraction-limited microscopy

### smFRET for mechanistic studies of MTs

**Fig. 5:**
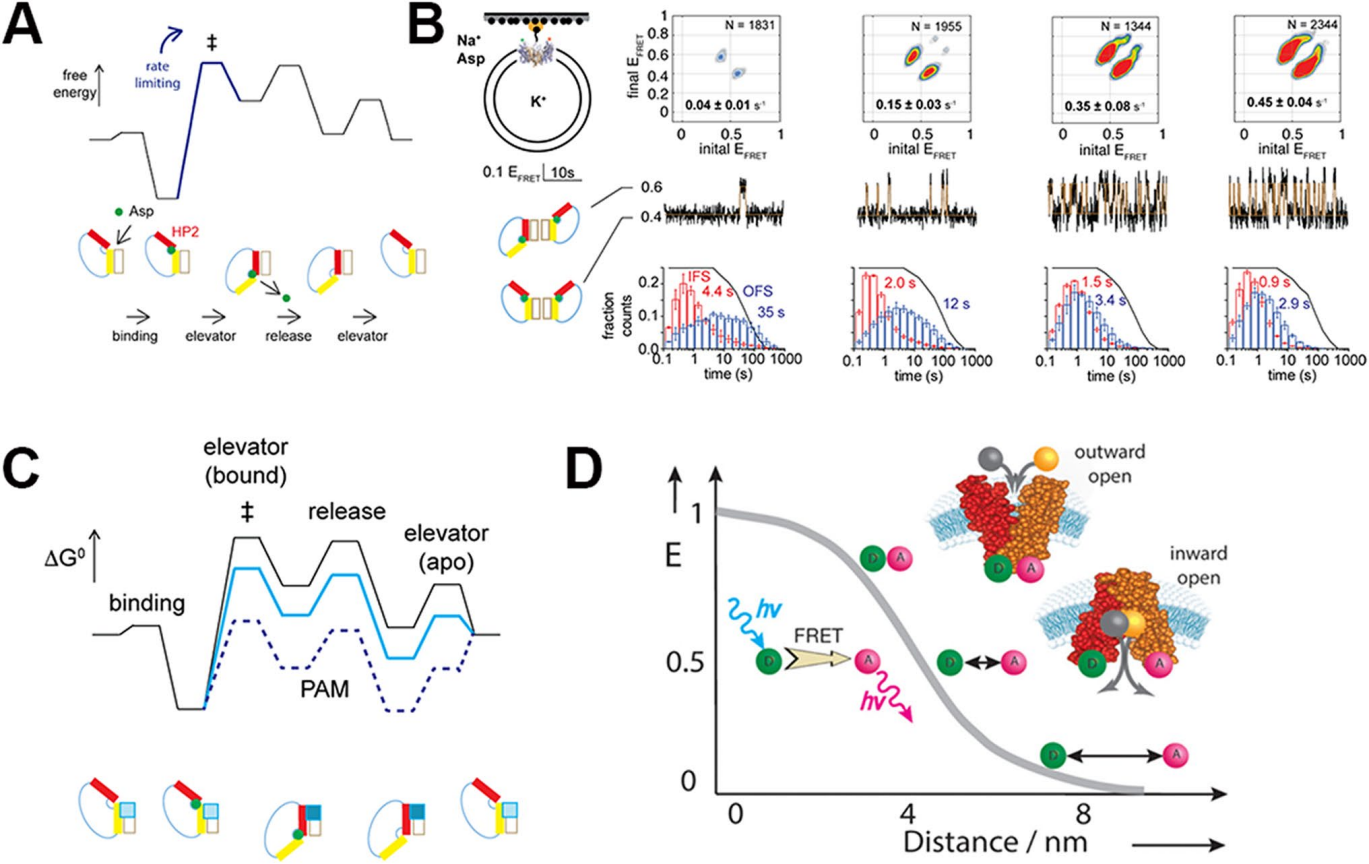
smFRET can be used to study features of a transport protein. **B** A stylised model of the rearrangements of the HP1 (yellow) and HP2 (red) domains within GltPh upon binding to aspartate (green) and the transition through the ‘elevator’ rearrangement in the transport steps. Shown above, this is the free energy changes associated with each step. The transition state formation step ‡ is energetically rate limiting. In the experiments reproduced in **B**, Huysmans and colleagues followed the movement of the transport domain within GltPh between the OFS and IFS by smFRET, using TIRF microscopy. To achieve this, they labelled a single cysteine residue within the transport domain self‐healing fluorophores (Akyuz et al. 2015) LD555P‐MAL and LD655‐MAL and biotin–polyethylene glycol–maleimide. The transporter was then reconstituted into proteoliposomes and immobilised using a streptavidin–biotin bridge in microfluidic perfusion chambers to facilitate rapid buffer exchange. A schematic of this is shown on the left. Data are shown for wildtype GltPh and selected gain‐of‐function mutants, as indicated above the panels. Transition density plots (top panels) show the frequency of transitions between the EFRET values. The number of trajectories analysed (N) and the population‐wide mean frequency of transitions are shown on the Panels. Representative 50‐s sections of single‐molecule EFRET trajectories are shown in the middle panels (raw data in black and idealisations in brown). Scale bar and the conformational states corresponding to the low and intermediate EFRET values are shown on the left of the panels. Dwell‐time distributions for the OFS in blue and the IFS in red are in the bottom panels. These experiments show conditions in which both Na+ and aspartate are on the outside of the proteoliposome and K+ on the insude. These can be varied—see Huysmans citation for details. Using this system, the authors are able to monitor the relative movements of the donor and acceptor-labelled domains at 100‐ms time resolution. **C** How the energetic landscape shown in **A** would change with a positive allosteric regulator (blue box). **D** How smFRET could be adapted to study domain movements within single transporters using site specific donor (**D**) and acceptor (**A**) labelling. A, B and C are reproduced from Huysmans et al. (2021) and D from Isselstein et al. (2020) under http://creativecommons.org/licenses/by-nc-nd/3.0/

A combination of advances in structural determination methodologies and predictive tools has resulted in a considerable number of structures of membrane transporters being available, showing transporters in different conformational states and allowing identification of, for example, substrate binding sites within these structures (Jumper et al. 2021). These studies, while incredibly important and informative, do not reveal details of the molecular transitions which underpin the kinetic mechanisms, nor how these high-energy transition states may be impacted by substrates, inhibitors or other (post translational) modifications of the transporter. Glt_Ph_ is an aspartate transporter isolated from *Pyrococcus horikoshii* which uses a transmembrane sodium gradient to transport one aspartate and three sodium ions across the membrane and has been extensively studied using structural biology and molecular dynamics simulations. A so-called ‘elevator movement’ of the transport domain of Glt_PH_ from an outward-facing (OFS) to an inward-facing (IFS) conformation is responsible for the movement of the solutes across the membrane. This movement involves two pseudo-symmetrical helical hairpins (HP1 and HP2; see Fig. 5a).

More recently, detailed insight into this transporter has been provided using single-molecule FRET (smFRET) which has allowed study of specific residues or domains within the transporter via introduction of position-specific labels (see for example (Shen et al. 2017; Simon et al. 2024)). These studies, for example, showed that the translocation of the substrate‐loaded transport domain from the OFS to the IFS is the rate limiting step of the cycle (Akyuz et al. 2013, 2015; Ji et al. 2016). One striking example of the power of smFRET comes from a recent study by Huysmans et al. (2021). The authors used smFRET to understand the structure of the high-energy transition state that sits kinetically between the OFS and IFS states and used this insight to pinpoint gain-of-function mutations in HP2. Interestingly, they report that mutations in the transport domain can affect multiple steps of the transport cycle. In this manner, they note that using these kinds of structural analyses together with predictive modelling tools could result in the development of novel allosteric modulators: they note that an allosteric modulator that binds with high affinity for the IFS would likely bind more tightly to the transition state and thus reduce the energy barrier for the (rate limiting) elevator transitions, and so could be considered positive allosteric activators. Similarly, moieties that preferentially bind the OFS would increase the energetic barrier and serve as negative allosteric modulators. Modulation of human excitatory amino acid transporters could have considerable therapeutic potential (Fontana 2015), and the kinds of molecular insight developed from these single-molecule studies should allow a more rationalised approach.

The authors specifically study the aspartate transporter Glt_Ph_; however, they note that their methodology would support the study of any membrane transporter modified with FRET paired fluorophores on the transporter domains of the protein (Bartels et al. 2021). In this way, they note that the approach ‘conceptualizes how the rate‐limiting transition state of a transporter can be characterized structurally, informing on potential druggable sites to develop allosteric modulators, and provides a method to investigate the kinetic effects of post‐translational modifications’. It should also be noted that these studies, using an ancient organism, exemplify how these SMLM approaches are not limited to any particular kingdom of biology.

### MT intracellular trafficking

Fujita et al. (2010) studied whether GLUT4 trafficking is directly altered by insulin stimulation in live fat cells using quantum dot nanoparticle trafficking. This approach allowed for molecular detail to be resolved from a diffraction-limited confocal microscope. These studies revealed varied GLUT4 trajectories across a 10-s imaging window at speeds of 30 frames/s. By linear fitting to the trajectories and comparing insulin-stimulated and non-stimulated fat cells, Fujita et al. observed that insulin increased the velocity of GLUT4 in a time-dependent manner but that the movement of GLUT4 was slower than that of the transferrin receptor measured in the same way, indicating a dynamically different behaviour of these proteins, consistent with their distribution within distinct intracellular compartments. This approach allowed the authors to derive a multiparameter model, an immobile and mobile fraction of total GLUT4 identified in the absence of insulin, and that the fraction of immobile GLUT4 significantly decreased under insulin stimulation. This was recently extended by the same group who developed a two-colour quantum dot labelling approach to study the behaviour of GLUT4-positive and transferrin receptor-positive compartments within different subcellular regions of fat cells (Hatakeyama et al. 2022). This approach led to a refinement in the understanding of GLUT4 delivery to the cell surface by showing pronounced regional variations in the trafficking of these compartments: trafficking appears faster at the cell periphery than deep within the cell. Whether this is dependent on cell architecture/crowding or could reflect distinct subcellular microrheological differences remains to be determined. Regardless, these studies introduce a methodologically simplified approach to tracking individual molecules throughout the entirety of living cells utilising a conventional microscope system. Their results reveal new insights into GLUT4 trafficking, but the method is highly applicable to the study of many other single membrane proteins.

**Fig. 6:**
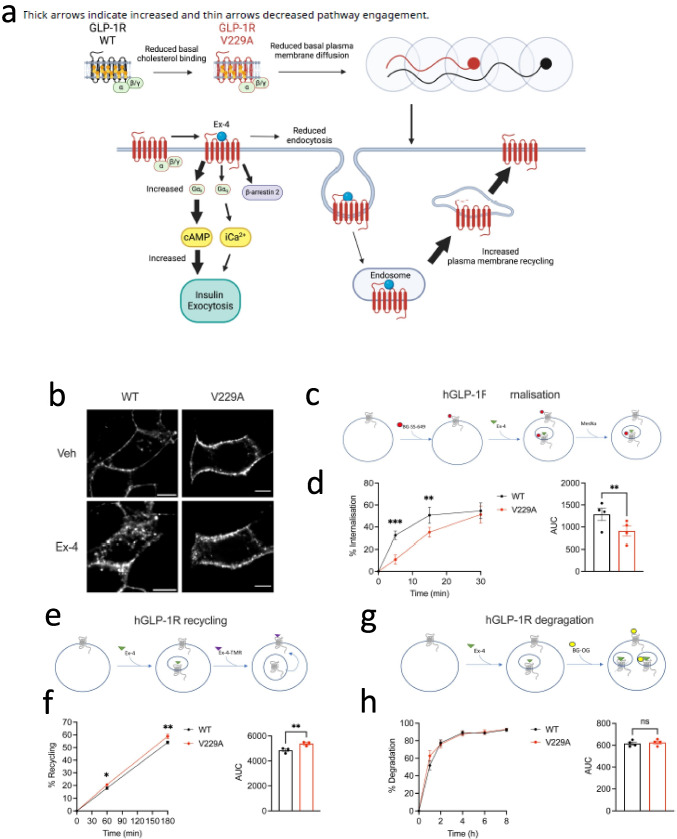
Visualising interaction between heightened cholesterol and GLP-1 receptor in vivo pancreatic beta cells. **a** Proposed model of interaction between GLP-1 receptor and cholesterol and the effects on signalling pathways following modulation of GLP-1/cholesterol interaction. **b**–**h** Effect of mutating GLP-1 cholesterol binding site on native trafficking of receptor. **b** Representative images of insulin stimulate GLP-1R wildtype labelled beta cells or mutated (V229A) cells labelled with SNAP-Surface Alexa Fluor 647, scale bars 5 µm. **c** Schematic of internalisation of labelled GLP-1 receptor. **d** Percentage of internalised WT vs V229A GLP-1 at time points past stimulation with 100 nM extendin-4, corresponding AUC also shown; *n* = 4. **e** Schematic of GLP-1 plasma membrane recycling assay. **f** Percentage of recycled WT vs V229A GLP-1 at time points past stimulation with 100 nM extendin-4. **g** Schematic of degradation assay. **h** Percentage of degraded WT vs V229A GLP-1 at time points past stimulation with 100 nM extendin-4, AUC also shown; *n* = 4. Data is mean ± SEM; ns, non-significant, *p < 0.05, **p < 0.01, ***p < 0.001 by paired *t*-test or two-way ANOVA with Sidak’s multiple comparison test. Reproduced from Oqua et al. (2024) under Creative Commons Licence CC BY 4.0

As discussed above for the work of Buenaventura et al. (2019) (Sect. ‘Understanding Wegovy’), the lipid environment of a cell PM is of pivotal importance to the function and organisation of critical G-protein coupled receptors (GPCRs), such as the GLP-1 receptor, which has been exploited for weight loss and management of type-2 diabetes. Utilising in vivo SPT of cAMP sensors, FRET imaging and Raster Image Correlation Spectroscopy (RICS), Oqua et al. (2024) studied the effect of heightened cholesterol in the regulation of GLP-1R, the interaction between the receptor and the lipid, and the potential role their interaction holds for subtle modulations of the response of GLP-1R. This impressive study builds on previous work in this research group elucidating the interaction of cholesterol and GLP-1R and an identified modulation of the function of GLP-1R in pancreatic beta cells by cholesterol. Both in vivo and in primary pancreatic islets, the authors observed a reduced glucose regulation via GLP-1R when cholesterol levels were raised. However, in the reverse experiment of pharmacologically lowering cholesterol levels and observing the effect on the GLP-1R, the authors observed that a drastic lowering of cholesterol (via treatment with methyl-β-cyclodextrin (MβCD)) was also deleterious, disrupting lipid architecture and impacting GLP-1 function. Following molecular dynamics simulations, they identified a potential cholesterol binding site on the receptor, which was mutated for this study. The colocalisation between labelled cholesterol and GLP-1R significantly decreased upon mutation, with a corresponding decreased displacement and speed of GLP-1 measured via RICS. There was a marked effect of GLP-1R trafficking upon mutation; receptor internalisation under insulin stimulation slowed in the short term but recovered after 30 min in the presence of the agonist extendin-4 (Fig. 6). Further, this mutation affected a host of signalling pathways (cAMP, insulin, other GPCRs) as summarised in Fig. 6a. Taken together, the work of Oqua et al. shows the critical role cholesterol has in the function of GLP-1R; how an elevated cholesterol level may have an adverse effect on patients with type-2 diabetes who are already on cholesterol management drugs such as statins; and how these treatments may be complemented by GLP-1 agonists to fine-tune glucose regulation by these receptors.

## Discussion

From the wide range of research articles selected for discussion in this review, it is clear that optical microscopy as a field has played an important role in revealing novel organisation, diffusion parameters and protein-protein interactions for a host of different membrane transporter proteins. Methods capable of spatially resolving single molecules have revealed clustered nanodomains of molecules in the PM of model membranes to prove concept of a polarisation-coupled PAINT microscopy and of the much-discussed GLP-1 receptor in the PM of pancreatic beta cells, to provide a better understanding of the signalling pathways of this critical type-2 diabetes implicated molecule. SMLM methods have been adapted to couple molecular orientation into localisation coordinates, with a simplified experimental setup to provide ease in biological measurements. Novel regulatory mechanisms across critical receptors in neuronal water transporters, glucose transporters, transporters facilitating waste clearance and inflammation regulation have been revealed utilising SMLM methods.

Hardware and analysis tools for SMLM are becoming more commonplace in recent years, and commercial microscopes are being routinely brought to market with more sophisticated SMLM modalities available to users. However, as the “Section 3: obtaining molecular resolution from a diffraction-limited microscope” and “Section 4: MT studies utilising diffraction-limited microscopy” sections of this review discuss, detail on the trajectories and interactions of single molecules can be revealed via a diffraction-limited microscope by utilising the photochemistry of FRET fluorophores and the high temporal resolution of a widefield, diffraction-limited microscope. Scientists have used these systems to identify transport of solutes across a membrane transporter, tracked a critical glucose transporter *in situ* in real time and observed the dynamic properties of GLP-1R, yielding a wealth of biological and biophysical detail from optically conventional microscope systems. From the “Section 4: MT studies utilising diffraction-limited microscopy” section, it is clear that multimodal single-molecule and diffraction-limited imaging is becoming routine in biophysical studies, coupling, for example, FRET with TIRF illumination or correlation spectroscopy methods with SPT. As with all scientific methodology, there is not a ‘one size fits all’ for all biological imaging, and the application of multiple techniques in tandem or in sequence is oftentimes the most suitable approach for answering a given research question.

It must be noted that while this review has focused on light microscopy methods for studying single membrane transporter molecules in situ, these are not the only biophysical methodologies capable of these studies. Atomic force microscopy has a significantly superior spatial resolution than light microscopy, which has been extensively used in this field (Tyagi et al. 2011; Pan et al. 2018; Deniz et al. 2008; Hodel et al. 2021; Unsay and García-Sáez 2019; Bronder et al. 2016). Indeed, as noted earlier in this review, while electron microscopy does require stringent and often destructive sample preparation, it still remains the gold standard in spatial resolution, and there has been a push towards correlative approaches across bioimaging in recent years (Peddie et al. 2017; Bushby et al. 2012; Brown et al. 2012; Boer et al. 2015). This is particularly evident within the UK and Eire, where there is strong expertise and an open community in EM and CLEM approaches, allowing a researcher to elucidate further insights from their specimens by firstly imaging with their chosen fluorescence microscopy method and then preserving and observing finer detail in EM.

Further, beyond the diffraction-limited and single-molecule localisation studies discussed in this review, many new insights can be gleaned from a specimen by utilising novel biochemical probes. As an example, novel membrane tension fluorophores have been designed where a mechanical change in PM architecture will result in a polar change to an embedded Flipper mechanosensitive probe which can be mapped as a change in fluorescence lifetime using fluorescence lifetime imaging microscopy (FLIM) (Roffay et al. 2024). This highlights an aspect of science on which the field of microscopy itself has benefitted: advancements in our understanding arise from the complementary developments of connected fields of study. In order to be in this current space of observing single molecules and their native interactions, development of chemical probes, advanced instrumentation, understanding and exploitation of complex photophysics and biochemical studies of these interesting single molecules was required and accounts for many decades of scientific research.

The advanced imaging methods discussed in this review have improved our understanding of the organisation and function of MTs implicated in neuronal signal transmission and water transport, insulin signalling, glucose trafficking, pyroptosis and protein oligomerisation. While all elucidate detail on very different molecular transporters, in every case, the experimental pipelines developed are highly applicable to any molecule of interest. The limiting factor across the board in these studies is a question of scale and broader implications of results observed in situ in cell membranes across the whole organism. Throughput of optical methods capable of resolving molecular detail is generally low, and population results are therefore limited.

With the great, and ever-growing, variety of advanced optical microscopy methods available to the researcher, the techniques on which a research group will hone in on and develop expertise in will generally depend on practical considerations such as expenditure, institutional availability and accessibility of training. However, as a field more broadly, we would expect that the cutting-edge SMLM method of Exchange-PAINT and structured super-resolution MINFLUX methods to be at the forefront of novel studies of MTs. Exchange-PAINT (Jungmann et al. 2014) is a technique capable of multiplexing to the *n*-th degree (where *n* is set by the number of molecules of interest one could feasibly afford the cost of DNA-oligomer reagents and hardware to appropriately sample n channels). MINFLUX/STED (Yu et al. 2019), the perhaps slightly more advanced successor to the well utilised STED microscopy, is capable of a similar spatial resolution to SMLM while retaining a much greater temporal resolution than its SMLM counterparts. A broader, and more clinically relevant, application of these methodologies may lie in the hands of the biomedical industry, coupling single-molecule imaging technologies with high-throughput screening and potentially with transcriptomic or metabolomic approaches to quantify molecular changes across whole organisms and tissues in a physiologically meaningful manner, lending itself well to drug discovery and therapeutic developments.

Together, the discussed single-molecule imaging studies represent the advancement of our understanding of a large array of membrane transporters implicated in health and disease, while the imaging approaches generally could be applied to study any molecule of interest. This demonstrates the power of biophysical imaging, which stands on the centuries of physical and chemical development of microscopy since the establishment of the field by Leeuwenhoek and Hooke.

## Data Availability

No datasets were generated or analysed during the current study.

## Abbreviations

AFM: Atomic force microscopy
AMPA: α-Amino-3-hydroxy-5-methyl-4-isoxazolepropionic acid
AQP: Aquaporin
ATP: Adenosine triphosphate
cAMP: Cyclic adenosine monophosphate
CNS: Central nervous system
CLEM: Correlative light and electron microscopy
DCC-SMLM: Dual-colour colocalisation SMLM
DNA: Deoxyribonucleic acid
DoLP: Degree of linear polarisation
EM: Electron microscopy
FLIM: Fluorescence lifetime imaging
FRET: Förster (fluorescence) resonance energy transfer
GABAaR: Gamma-aminobutyric acid type A receptor
GLUT: Glucose transporter
GLP-1R: Glucagon-like peptide-1 receptor
GltPh: Glutamate transporter homologue from *Pyrococcus horikoshi*
GlyR: Glycine receptor
GP: Generalised polarisation
IR: Insulin receptor
ISO: Isoproternol
MβCD: Methyl-β-D-cyclodextrin
MDGA: MAM domain-containing GPI-anchored protein
MINFLU: Minimal photon fluxes
MT: Membrane transporter
PAINT: Point accumulation in nanoscale topography
PALM: Photo-activated localisation microscopy
PM: Plasma membrane
POLCAM: Polarisation camera SMOLM
RICS: Raster image correlation spectroscopy
SBR: Signal-to-background ratio
SMLM: Single molecule localisation microscopy
SMOLM: Single molecule orientation localisation microscopy
SPT: Single-particle tracking
STED: Stimulated emission detection
STORM: Stochastic optical reconstruction microscopy
SV2A: Synaptic vesicle protein-2A
Syt1: Synaptotagmin-1
TIRF: Total internal reflection fluorescence
